# DNA barcoding reveals global and local influences on patterns of mislabeling and substitution in the trade of fish in Mexico

**DOI:** 10.1371/journal.pone.0265960

**Published:** 2022-04-14

**Authors:** Adrian Munguia-Vega, Renata Terrazas-Tapia, Jose F. Dominguez-Contreras, Mariana Reyna-Fabian, Pedro Zapata-Morales

**Affiliations:** 1 Conservation Genetics Laboratory & Desert Laboratory on Tumamoc Hill, The University of Arizona, Tucson, Arizona, United States of America; 2 Applied Genomics Lab, La Paz, Baja California Sur, México; 3 Oceana en México, Protegiendo los Oceanos del Mundo A.C., Mexico City, México; 4 Instituto Politécnico Nacional–Centro Interdisciplinario de Ciencias Marinas (IPN- CICIMAR), La Paz, Baja California Sur, México; 5 Departamento Académico de Ciencias Marinas y Costeras, Universidad Autónoma de Baja California Sur, La Paz, Baja California Sur, México; Universita degli Studi di Bari Aldo Moro, ITALY

## Abstract

Mislabeling of seafood is a global phenomenon that can misrepresent the status and level of consumption of wild fish stocks while concealing the use of many other wild species or those originating from aquaculture and sold as substitutes. We conducted a DNA barcoding study in three cities within Mexico (Mazatlan, Mexico City and Cancun) and sequenced the COI gene in 376 fish samples sold as 48 distinct commercial names at fish markets, grocery stores, and restaurants. Our goal was to identify the main species sold, their mislabeling rates and the species most used as substitutes. Overall, the study-wide mislabeling rate was 30.8% (95% CI 26.4–35.6). Half of the samples collected belonged to five species traded globally (yellowfin tuna, Atlantic salmon, mahi, swai, and tilapia), most of them with important aquaculture or ranching production levels. These species were commonly used as substitutes for other species and showed low mislabeling rates themselves (≤ 11%, except mahi mahi with 39% mislabeling). The other half of the samples revealed nearly 100 species targeted by small-scale fishers in Mexico and sold under 42 distinct commercial names. Popular local commercial names (*dorado*, *marlin*, *mero*, *robalo*, *mojarra*, *huachinango*, *pargo*, *sierra*) showed the highest mislabeling rates (36.3% to 94.4%) and served to sell many of the 53 species identified as substitutes in our study. We discuss the observed patterns in relation to landing and import data showing differences in availability of commercial species and the links to explain observed mislabeling rates and the use of a species as a substitute for other species. We also outline some of the implications of establishing a labeling and traceability standard as an alternative to improve transparency in the trade of seafood products in Mexico.

## Introduction

Global demand for seafood is at an all-time high and is predicted to keep growing significantly in the near future [1, 2]. While nearly 60% of assessed wild fish stocks are fully exploited with no room for additional fishing pressure and 30% are overfished and in need of rebuilding [3], it is increasingly relevant to understand which wild fish species are used to feed people around the world to avoid overfishing of their populations. Alongside, as aquaculture production recently surpassed the volume of wild-capture fisheries [3], aquaculture species could start replacing wild fish in our diets, particularly from overfished species showing high demand but low availability. Although tracking the identity and origin of wild and aquaculture seafood products during their commercialization has become necessary, it also represents a considerable challenge due to mislabeling and substitution. DNA barcoding using the COI gene [4] has been widely used as a reliable and accessible technique for species identification and delimitation in a wide array of taxa [5, 6].

The persistence of seafood mislabeling has been well documented, varying between 30% of mislabeled samples across 51 peer-reviewed reports [7] to 19% mislabeling among 200 studies [8] to a recent estimate of 24% among 141 studies [9]. One emerging pattern is that, although mislabeling can occur for virtually any species, some species seem to be much more prone to mislabeling than others that are rarely substituted [10, 11]. Mislabeling is usually attributed to multiple drivers, including economic fraud, commercialization of illegal, unreported and unregulated (IUU) products, or unintentional misidentification due to poor traceability [12, 13]. Substitute species are commonly of lower value than those listed on labels and menus [11]. Mislabeling is also one way IUU products enter the supply chains [14, 15], including the commercialization of threatened species such as sharks and rays [16–18].

An often-overlooked aspect of seafood mislabeling is how it can misrepresent the actual status of wild fish stocks, signaling the abundance of a species that appears to be plentiful in the market but that in reality is scarce due to overfishing and thus is substituted by other species. This effect referred to as “dilution”, occurs when declines in supply from individual fisheries are hidden from consumers through substitution with alternative species, including those from aquaculture [19]. Understanding the dilution effect requires the classical approach of explaining which species are mislabeled while looking at the complementary question of which species are used as substitutes [20]. Under this rationale, mislabeling could be explained as a balance between supply and demand of species in the market, where species with high demand and low supply can be expected to show higher levels of mislabeling and rarely be used as substitutes. In contrast, species with low demand and high supply are expected more commonly to be used as substitutes but show low rates of mislabeling. It has also been suggested that substituted products worldwide come from fisheries with less effective management, less healthy stocks and greater impacts of fishing on other species [21].

Numerous studies have documented seafood mislabeling, with 70–80% of these coming from the USA/Canada and the European Union [7, 9, 22], representing some of the largest markets driving global seafood demand. However, fewer studies exist about seafood mislabeling from developing countries, from which the majority of the global seafood supply originates [3]. The dynamics of seafood mislabeling (and the strategies to reduce it) vary between developed and developing countries in multiple ways. Developing countries with lower incomes usually show higher levels of biodiversity [23, 24], stronger reliance on small-scale fisheries in terms of livelihood support at the microeconomic level [25], and varying degrees of regulatory frameworks, monitoring and enforcement capacity [26], among others. Studies in the global north usually define mislabeling as when a particular species is advertised or sold under a different name than an official register that matches scientific names to commercial names under which they can be sold. For example, the Seafood List published and regularly updated in the US by the Food and Drug Administration [27]. In contrast, many countries from the global south (including Mexico) lack such official list, and other unofficial criteria matching scientific and commercial names need to be used to establish when mislabeling occurs.

In Latin America, seafood mislabeling studies have been conducted in Belize [28], Costa Rica [29], Chile [30], Peru [23] and Brazil [31–34]. According to a recent study that estimated the seafood consumption footprint for 100 countries in 2011 (the biomass of domestic and imported seafood production required to satisfy national seafood consumption), Mexico’s consumption (1.32 million tons) is the second largest in Latin America after only Brazil [1]. However, studies of seafood mislabeling in Mexico have been limited [35, 36]. Only two studies with a large sampling scope exist from Mexico. A study from fish markets in Mexico City, Gulf and Caribbean coasts of Mexico found 18% mislabeling and the commercialization of threatened species of bony fish and sharks according to the IUCN Red List criteria [37]. A recent study focused on small-scale fisheries reported 40% mislabeling in fish markets and restaurants from La Paz, Mexico [20].

A key concept commonly invoked regarding seafood sustainability is the urgent need to develop traceability schemes that can record the history of seafood from sea to table [38]. Seafood traceability refers to the ability to access all information about a seafood product throughout its entire life cycle, using recorded identifications [39]. Traceability is also linked to food safety, socioeconomic benefits to fisheries transparency, business efficiency, quality control and compliance with international law. Most important, seafood traceability is a crucial tool to reduce IUU fishing [40].

Mexico lacks a seafood traceability system in the food safety and fisheries management sectors. The Mexican regulation on food and safety of seafood products establishes the “one step forward, one step back” approach, which has the primary purpose of removing contaminated products when they are a health hazard to consumers. When a hazardous product is identified, Mexican authorities track its origin by asking the owner of the product of whom it was bought, and they keep that chain until they find the origin of the product and request the removal of all of it. As for proof of the legal origin of seafood products, Mexican law requires different documents for different parts of the process. Fishers, for example, are required to have a valid fishing permit or a fishing concession. Once they have arrived at a port, they must report their catch to the fishing authorities and receive a landing slip in return. If the fisher, or whoever purchased the catch, intends to transport the product across state boundaries, they require a transport permit (guía de pesca). From there, it only gets more complex, as each step of the supply chain requires at least a photocopy of the documents that verify the previous steps, even if, as it often happens, a truckload is made up of the products of different catches on different days. Different rules apply to the trade of threatened and protected species. In reality, rules are often applied subjectively and inconsistently, and the burdensome regulatory framework offers ample opportunities for corruption.

The goal of our study was to describe the nature and frequency of mislabeling of fish across three different types of vendors (fish markets, grocery stores, and restaurants) in three main cities of Mexico to expand the understanding of the practice of mislabeling in Mexico. We sought to answer the following questions: 1) Which main fish species are sold commercially and under which names? 2) What are the mislabeling rates for the most frequently used commercial names? 3) Which species are most used as substitutes and their origin? 4) Are there any trends in mislabeling commercial names and the use of certain species as substitutes concerning the net availability of species in the market?.

## Methods

### Sampling

We collected 462 commercial fish samples from July-September 2018 in three Mexican cities, Mazatlan (a large city in the Pacific state of Sinaloa), Mexico City, and Cancun (a large city in the Caribbean state of Quintana Roo). Within each city, sampling focused on three distinct types of vendors: restaurants, grocery stores, and fish markets, which represent the three main points of sales where regular consumers buy fish. We selected fish vendors based on criteria to maximize the samples’ representation. We obtained a list of restaurants that sold fish dishes within each city using the application TripAdvisor. Then, we categorized each restaurant into two distinct price categories (upper and lower, respectively), based on the range prices provided by the application, prices obtained from each restaurant webpage and prices confirmed upon visit for sampling. We used a cutoff of 10 USD per dish selling fish (200 MX Pesos) to classify each restaurant into two categories. We sampled at least once all the major chains of grocery stores present within each city that sold fresh or frozen fish products. For fish markets, we focused our sampling on the main commercial hubs for buying/selling fish where multiple vendors are commonly present next to each other, including temporary markets that are established directly in the street or permanently within a building. We also obtained the location of fish markets by searching within the application Google Maps.

In Mazatlan, we obtained samples from 23 restaurants, seven supermarkets and 11 fish markets; in Mexico City, from 26 restaurants, eight supermarkets and 21 fish markets; and in Cancun, from 21 restaurants, seven supermarkets and nine fish markets. We divided our sampling efforts equally between restaurants from each city’s upper and lower price categories. We purchased the samples acting as regular and anonymous restaurant clients and fish buyers at fish markets and grocery stores. Sampling was not focused on any species and included as many different commercial names were available by each vendor to increase representation. Each sample (approx. 0.5 grams of fish tissue) was collected and preserved in screw-cap 2 ml tubes containing silica beads. For each sample collected, we registered the following information on a custom-made phone application (https://www.zoho.com/forms): Unique ID; vendor name and category (fish market, grocery store, restaurant); the commercial name of the fish as provided by the vendor; source of the commercial name (label, menu, verbal communication); type of sample (fresh, frozen, fried, grilled, breaded, dry); the price per kilogram (fish markets and grocery stores) or portion (restaurants); and additional information or comments.

### Genetic identification

We extracted genomic DNA with a modified salting-out protocol [41]. For 137 samples, the salting-out protocol produced low-quality/quantity genomic DNA. We repeated the DNA extraction with a DNeasy blood and tissue kit (QIAGEN) for these samples. We amplified via the Polymerase Chain Reaction (PCR) ~655 bp of the Cytochrome Oxidase subunit I (COI) employing primers and protocols previously reported [42]. We verified successful PCR amplification on 1.3% agarose gels stained with GelRed (Biotium) and obtained forward and reverse sequences with an Applied Biosystems 3730XL Sanger sequencer. The resulting sequences were edited by eye to create a consensus sequence with the online software tool BENCHLING (https://benchling.com). We used the Clustal W algorithm in the software MEGA7 [43] to create a multiple alignment of the sequences and verify they contained uninterrupted open-reading frames characteristic of a functional protein.

We obtained genetic identification of the edited sequences comparing against two databases: 1) NCBI nucleotide database with the Blast-n search tool [44] using the Megablast algorithm for highly similar sequences; 2) the barcode of life database BOLD (http://www.barcodinglife.org), against the "species-level barcode records". Species identification followed the match to the most similar sequence present in each database with sequence similarity of at least 98%.

To establish mislabeling, we compared the commercial name provided by the vendor against three reference databases that contain commercial and scientific names of fish from Mexico: 1) an online catalog for fisheries species in the Pacific coast of Mexico (http://catalogo.cicimar.ipn.mx), which details the commercial (common) names for 924 marine species based on three sources: common names recognized by the Food and Agriculture Organization of the United Nations (FAO)_ in Spanish, the Mexico National Fisheries Chart and common names mentioned in other scientific references [45]; 2) a catalog of commercial marine fishes maintained by the National Biodiversity Commission (CONABIO) (http://enciclovida.mx/peces); 3) The list of common names in Spanish for Mexico supported by the open-access database Fishbase (http://www.fishbase.org). We considered a sample mislabelled if the commercial name provided by the vendor did not match the common name of the genetically identified species in any of the three catalogs above. All confidence intervals (CI, α = 0.05) around mislabeling rates were calculated using Wilson’s method. The relationships between scientific names and the 18 most important commercial names are shown in S1 Table.

### Mislabeling and substitutability

We followed a recently developed framework based on network analysis to describe how a particular species is mislabeled and used as a substitute to other species to estimate its net availability in our sampling (See Table 1 for detailed definitions of each term) [20]. We defined focal species as a particular commercial name for which mislabeling or substitution is being estimated.

**Table 1:** The terminology used in the analyses of seafood mislabeling and substitution, following Munguia-Vega et al. [2021].

Term	Explanation
Verbal sample number	Number of samples analyzed under the commercial name of the focal species, as communicated by the vendor.
Correctly labeled samples	Number of times samples sold as the focal species were correctly labeled.
Mislabeling frequency	Number of times samples sold as the focal species were mislabeled.
Mislabeling percentage	Percentage of mislabeled samples relative to the verbal sample number.
Mislabeling diversity	Number of different species sold under the name of the focal species. Used as a proxy for demand in our dataset.
Substitutability frequency	Number of times samples from the focal species were used as substitutes for other species. Used as a proxy for demand in our dataset.
Substitutability diversity	The number of different species that the focal species substituted. Used as a proxy for demand in our dataset.
Confirmed samples	Correctly labeled samples + substitutability frequency. This is the real number of samples genetically identified for the species associated with the commercial name, after considering mislabeling and the use of the species as substitute. Used as a proxy for the net availability of species in our dataset.
Over/sub-representation	Difference between the verbal sample number and the number of confirmed samples.
Percentage of over/sub-representation	Percentage of the difference between the verbal sample number and the number of confirmed samples.

From the number of samples analyzed under a particular commercial name as told by vendors (Verbal sample number, Table 1), we first subtracted the number of samples sold as the focal species that were mislabeled according to the genetic analyses as explained above (Mislabeling frequency) to obtain the number of samples that were Correctly labeled (Table 1). Then, to the Correctly labeled samples, we added the number of samples from the focal species used as substitutes for other species (Substitutability frequency) to obtain the number of Confirmed samples. The number of Confirmed samples represents the actual number of samples genetically identified for a particular species after mislabeling and substitution patterns are considered, and it was used as a proxy for the net availability of each species in our sampling.

We estimated substitutability as a proxy for the demand of a species in our dataset in three different ways following Munguia-Vega et al. [2021]. First, we used substitutability frequency as defined above, assuming that species in high demand will be less likely to be used as substitutes for other species, showing lower substitutability frequency. Second, we estimated the number of different species sold under the name of the focal species (Mislabeling diversity, Table 1) under the rationale that species with high demand would show higher mislabeling diversity values. Third, we calculated the number of different species that the focal species substituted (Substitutability diversity, Table 1). We expected that species with high demand if used as substitutes, would replace only a small number of other species and show small substitutability diversity values. We used linear regression analyses to test the relationship between our proxies of net availability and substitutability in our samples to predict the mislabeling rates of the 18 most common commercial names. To test if the mislabeling patterns observed were simply related to the frequency of each commercial name in the study and not specifically to the confirmed number of samples after genetic analyses, we also compared the verbal sample number from each commercial name against the observed mislabeling rate.

### Net availability from landing and import data

To test the hypothesis that the level of supply of particular species could help explain observed patterns of seafood fraud, we collected official information of landings (tons) from a national database (Aquaculture and Fishery Statistic Yearbooks) elaborated by the National Fisheries Commission in Mexico, CONAPESCA [46]. We obtained national landings data from 2010–2018 for species that were both frequent in our sampling and for which data was available from CONAPESCA at the level of identifiable species and commercial names, including s*ierra* (sierra), *mero* (grouper), *robalo* (snook), *huachinango* (red snapper), *atun* (tuna), and *mojarra* (mojarra)—this includes wild and aquaculture-. There are no official landing statistics for marlin since Mexican law restricts harvest of this species to recreational fishing and bycatch. Thus, in this case, we used the maximum allowed amount of *marlin* for both recreational and bycatch fishers according to official authorities during 2016, 2017 and 2018, including blue marlin (*Makaira nigricans*) and white marlin (*Tetrapturus spp*.) [47]. We used import records (tons) for basa and tilapia since only a very small percentage of the total supply of these is produced within Mexico. Data for other species (e.g., *dorado* or mahi mahi) was not available since it is merged in the official data, along with other ~250 species, in a category labeled as “others”. We tested the relationship between mislabeling percentage and availability from landings and import data from the year when we conducted our genetic sampling (2018) with linear regression. Because landing data was distributed over a difference of three orders of magnitude depending on the species, we log-transformed landing data before conducting analyses.

## Results

### Sampling and genetic identification

From 462 fish samples collected, we were able to amplify via PCR 416 samples whose PCR products were sent for sequencing. About half of the samples that failed to amplify were sold as smoked tuna or marlin and had a characteristic orange color suggesting the use of a colorant that might have interfered with PCR. We obtained quality DNA sequence data from 383 samples that averaged 553 bp, matched a fish species, and were clean and unambiguous. Most samples that were sequenced but excluded from analyzes matched bacterial DNA. Samples were collected in Mazatlan (N = 123), Mexico City (N = 153) and Cancun (N = 107). Overall, samples were purchased in 133 different commercial venues: 41 fish markets, 22 grocery stores and 70 restaurants. The resulting 383 sequences were deposited in GenBank (Accession numbers MN756096- MN756478, S2 Table).

Except for two sequences, all showed ≥ 98% homology with at least one sequence in the reference databases. The two exceptions were samples identified as *Cynoscion parvipinnis* (92.6%) and *Sciades seemanni* (94.5%), which likely correspond to other closely related species not represented in the reference databases, and their identification could be considered reliable only at the genus level. For most of the samples (356, 93%), both reference databases agreed to genetic identification of the same species. In the other 7%, both databases agreed only at the genus level, and samples were assigned to the species showing the highest identity similarity. For 11 samples, the COI barcode provided identical similarity values in GenBank and BOLD to different closely related species within the genus *Thunnus*, *Lutjanus*, *Merluccius*, *Dasyatis*, *Oreochromis* and *Peprilus*. For these samples, the identification included both closely related species (S2 Table).

### Species and commercial names found

Excluding seven samples sold under the generic name "pescado" (fish), the other 376 samples were sold under 48 different commercial names (S3 Table) and represented 103 genetically identified species (S4 Table). Most commercial names (26) were found exclusively in a single city within Mexico, while only nine and 13 commercial names were found in two and three cities, respectively (S3 Table).

Five genetically-identified species were found in the highest frequency and together represented almost half of all the samples (45.4%): *Thunnus albacares* (yellowfin tuna, 16.7%), *Salmo salar* (Atlantic Salmon, 8.6%), *Coryphaena hippurus* (mahi mahi, 7.8%), *Oreochromis niloticus* (tilapia, 6.3%) and *Pangasianodon hypophthalmus* (swai, 6.0%). The rest of the samples (55.6%) were represented by 95 species with a frequency between one and eight samples (≤ 2.1%) (S4 Table). Notably, about half (51) of all the distinct species found were represented by a single sample.

We observed some differences in the species genetically identified and most frequently sold among the three cities in Mexico and the commercial names used in each city. For example, the three most frequent species found in Mazatlan were yellowfin tuna (26 samples), mahi mahi (23), and Atlantic Salmon (8). In Mexico City, the most frequent species were tilapia (18), yellowfin tuna (18) and Atlantic Salmon (11), while in Cancun the most frequent species were yellowfin tuna (20), Atlantic Salmon (14) and swai (12).

### Mislabeling patterns

According to our criteria, we found 116 instances of samples that were considered mislabeled, which translated into a study-wide mislabeling rate of 30.8% (CI 26.4–35.6). Out of the 48 commercial names reported by vendors, we focused our analyses on 18 main commercial names that showed a verbal sample number ≥ 6 samples. These 18 commercial names represented 316 samples (84% of all samples, Table 2). This group of samples allowed us to estimate mislabeling rates with relatively higher accuracy based on larger sample size. High mislabeling rates (53.3–94.4%) were found in six commercial names, in decreasing order of mislabeling: *marlin* (marlin), *sierra* (sierra), *mero* (grouper), *huachinango* (red snapper), *robalo* (snook) and *curvina* (corvina). Intermediate mislabeling rates (33.3–40%) were recorded for seven commercial names, including *mojarra* (mojarra), *dorado* (mahi mahi), *pargo* (snapper), *cochito* (triggerfish), *lenguado* (flounder), *peto* (wahoo) and *trucha* (trout). Low mislabeling rates (5.1–11.1%) were found for four commercial names, including *tilapia* (tilapia), *atun* (tuna), *cazon* (shark) and *salmon* (salmon). The commercial name *basa* (swai) was the only one not showing any mislabeling at all (Table 2).

**Table 2:** Patterns of mislabeling for 316 samples sold under the 18 most common commercial names in Mexico, representing 84% of all the collected samples (See Table 1 for detailed descriptions of each term used in the column headers).

#	Commercial name in *Spanish* (English translation)		Verbal sample number	Correctly labeled samples	Mislabeling frequency	Mislabeling %	Mislabeling diversity	Substitutability frequency	Substitutability diversity	Confirmed samples	Over/sub-represen-tation	Over/sub-represen-tation %
1		*Atun* (Tuna)	58	52	6	10.34	4	15	4	67	-9	-13.43
2		*Salmon* (Salmon)	39	37	2	5.12	2	3	2	40	-1	-2.5
3		*Cazon* (Shark)	32	29	3	9.37	3	5	3	34	-2	-5.88
4		*Dorado* (Mahi mahi)	31	19	12	38.70	9	11	10	30	1	3.333
5		*Marlin* (Marlin)	18	1	17	94.44	5	0	0	1	17	1700
6		*Tilapia* (Tilapia)	18	16	2	11.11	1	6	6	22	-4	-18.18
7		*Mero* (grouper)	15	2	13	86.66	9	2	2	4	11	275
8		*Robalo* (Snook)	15	7	8	53.33	8	3	3	10	5	50
9		*Mojarra* (Mojarra)	15	9	6	40	3	1	1	10	5	50
10		*Huachinango* (Red snapper)	13	6	7	53.84	7	1	1	7	6	85.71
11		*Basa* (Swai)	12	12	0	0	0	8	3	20	-8	-40
12		*Pargo* (Snapper)	11	7	4	36.36	4	2	1	9	2	22.22
13		*Sierra* (Sierra)	9	1	8	88.88	7	3	3	4	5	125
14		*Curvina* (Corvina)	6	3	3	50	3	3	2	6	0	0
15		*Cochito* (Triggerfish)	6	4	2	33.33	2	3	2	7	-1	-14.28
16		*Lenguado* (Flounder)	6	4	2	33.33	2	0	0	4	2	50
17		*Peto* (Wahoo)	6	4	2	33.33	2	5	3	9	-3	-33.33
18		*Trucha* (Trout)	6	4	2	33.33	2	0	0	4	2	50

### Substitutability patterns

**Fig 1:**
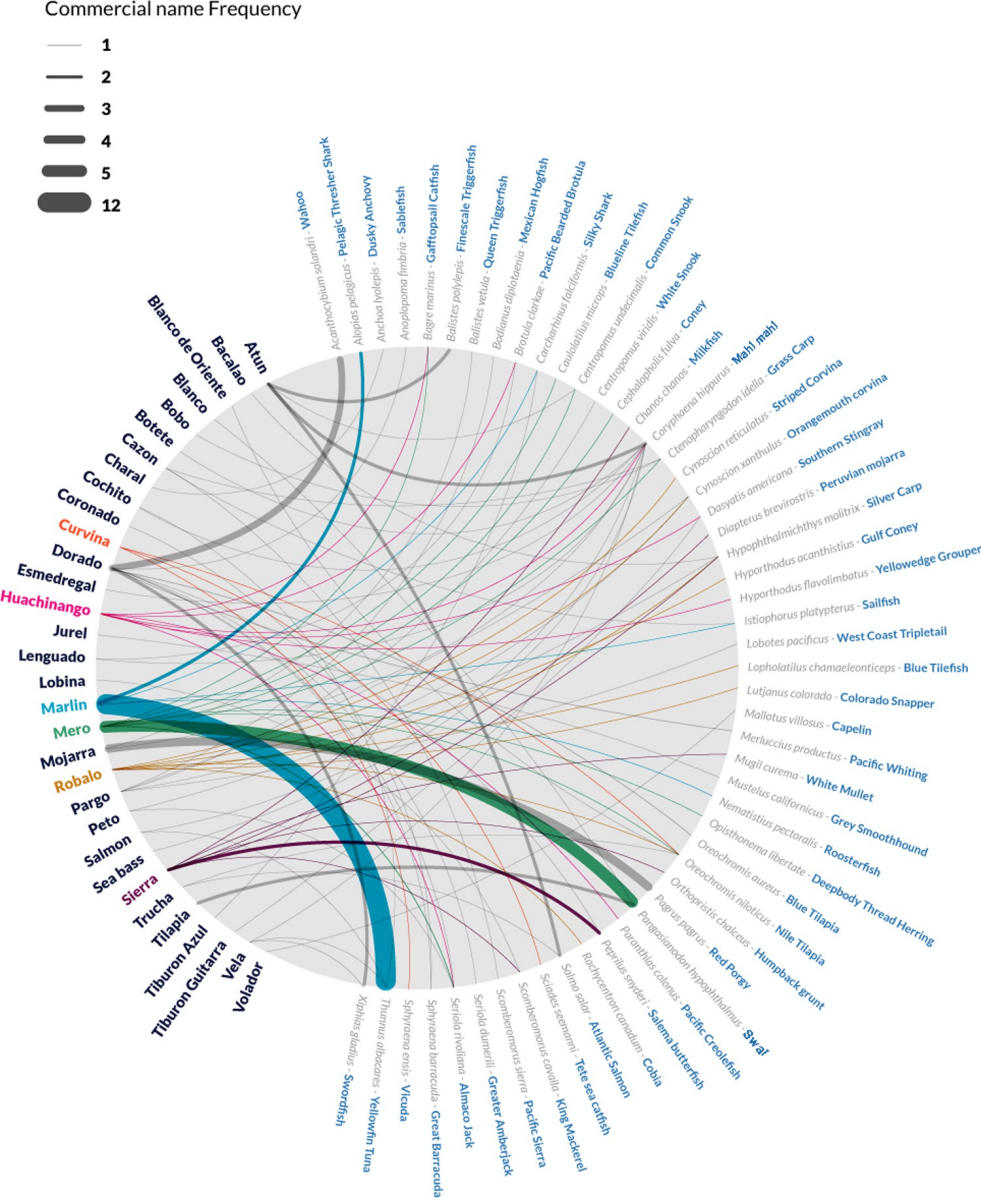
Alluvial plot showing the patterns of 116 instances of mislabeling found in Mexico, displayed as a network connecting 53 species used as substitutes for 32 commercial names that created 90 unique combinations under which samples were mislabeled. Line widths represent the frequency of a given mislabeling combination (thickest line = 12 events). The six commercial names with the highest frequency of mislabeling are shown with distinct colors.

Overall, 53 species were used as substitutes for 32 commercial names where mislabeling was found, representing a total of 90 mislabeling combinations as shown in Fig 1 and S5 Table. The resulting relationships depicted as a network in an alluvial plot were complex but contained within a single network. The four species most used as substitutes, which displayed the largest Substitutability frequency (Table 2), were in decreasing order of importance yellowfin tuna (12.9%), mahi mahi (9.4%), swai (7.2%) and tilapia (5.1%), followed by an extensive list of other 49 species used as substitutes with an observed frequency ≤ 4.3% (Fig 1, Table 2, S5 Table). The three species that substituted the largest number of commercial names, *i*.*e*., showed the highest values of Substitutability diversity (Table 2), were in decreasing order mahi mahi (substituted in 10 commercial names), tilapia (substituted in 6 commercial names), and yellowfin tuna (substituted in 4 commercial names). Around half of the 53 species used as substitutes (45.2%) substituted more than one commercial name (Fig 1, S5 Table).

Some species were clearly used as substitutes of a given commercial name with more preference than others. For example, yellowfin tuna substituted *marlin* 70.5% of the time, swai substituted *mero* 38.4%, and red porgy (*Pagrus pagrus*) substituted *mojarra* 100% of the time (Fig 1). The commercial names substituted by a greater number of species (*i*.*e*., with the largest values of Mislabeling diversity) were, in decreasing order *mero* and *dorado* each substituted by nine different species (respectively), *robalo* (substituted by eight species), *huachinango* and *sierra*, each substituted by seven species (Fig 1, Table 2). Around half of the commercial names (56.2%) were substituted by multiple species that ranged from two up to nine species.

### Mislabeling patterns: Cities, vendors, labels

Mislabeling rates showed some variation among the three cities examined, from 26.4% (C.I. 18.9–35.5) mislabeling in Cancun to 30.8% (C.I. 23.2–39.5) in Mazatlan, to 34% (C.I. 26.9–41.9) in Mexico City (Table 3) but differences were not significant (*X*^*2*^ ≥ 1.675 *P* ≥ 0.432). Overall, mislabeling rates were significantly lower in grocery stores (16.6%, C.I. 10–26.4) Table 3) compared to restaurants (33.5%, C.I. 27.2–40.4, *X*^*2*^ = 7.688, *P* = 0.005) and fish markets (36.4%, C.I. 27.9–45.8, *X*^*2*^ = 8.736, *P* = 0.003). Mislabeling rates were similar for labels and restaurant menus (17.7% C.I. 10.8–27.5, and 26.7% C.I. 19.8–35, respectively; *X*^*2*^ = 2.232, *P* = 0.135), but mislabeling decreased significantly when the source of the commercial name was a label as compared to a verbal conversation (40%, C.I. 32.9–47.5, *X*^*2*^ ≥ 12.120, *P ≤* 0.001).

**Table 3:** Patterns of fish mislabeling found in three cities within Mexico, including sample size (N) and mislabeling rates (% M).

	Mazatlan		Mexico City		Cancun		Total	
Vendor type	N	% M	N	% M	N	% M	N	% M
**Fish markets**	33	33.3	47	44.6	27	25.9	107	36.4
**Grocery stores**	24	8.3	33	24.2	21	14.2	78	16.6
**Restaurants**	63	38.0	70	31.4	58	31.0	191	33.5
**Total**	120	30.8	150	34.0	106	26.4	376	30.8

Few commercial names showed mislabeling rates that were consistently high among the different cities surveyed (≥ 50%, *e*.*g*., *marlin*, *robalo*, S6 Table) or consistently low (≤ 14.3%, *e*.*g*., *atun*, *salmon*, *basa*). Most commercial names showed contrasting patterns of mislabeling between cities (*e*.*g*., *dorado*, *cazon*, *tilapia*, *mojarra*, *huachinango*, *pargo*, *mero*, S6 Table).

### Mislabeling patterns: Marine bony fish, elasmobranchs and aquaculture

**Fig 2:**
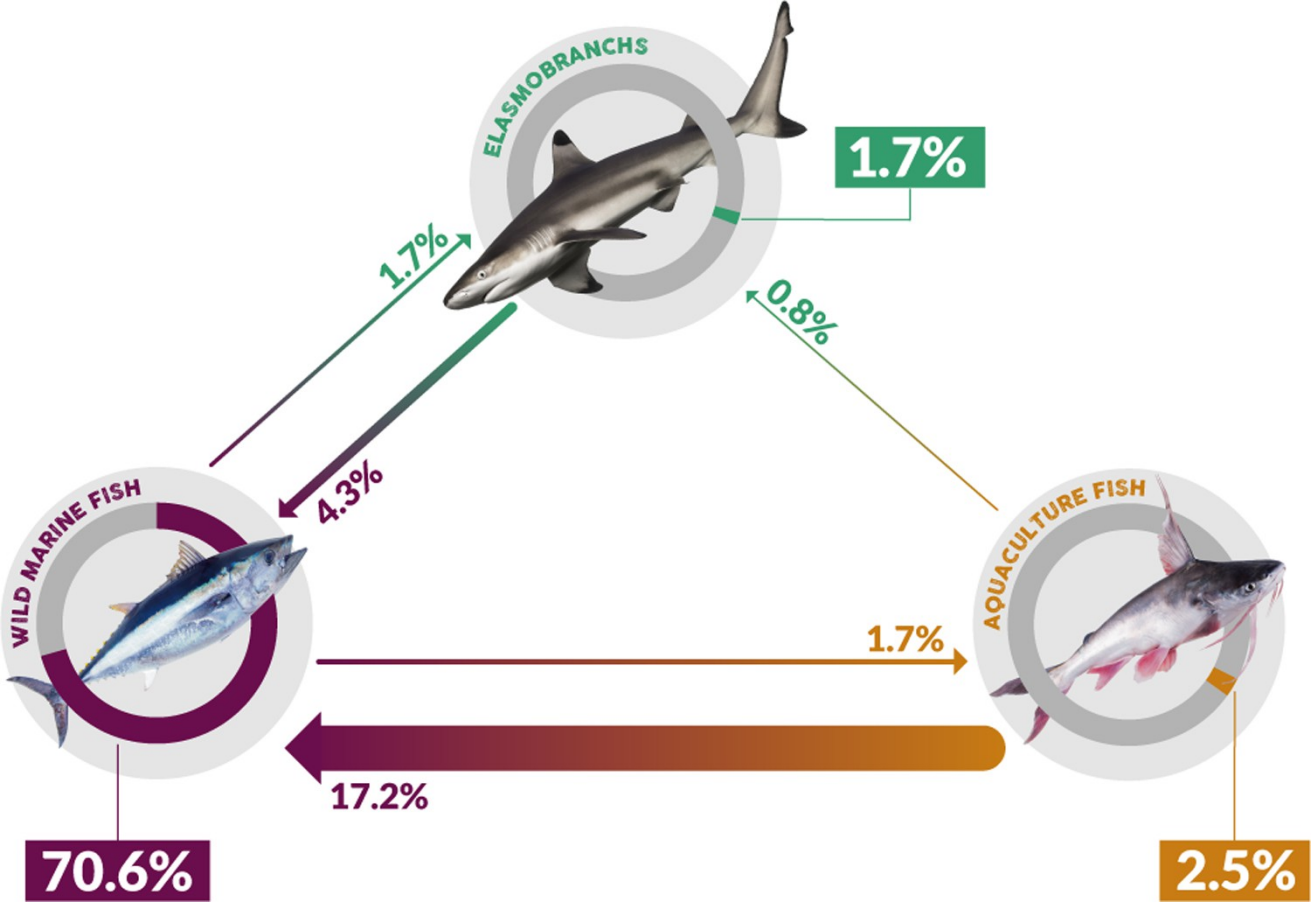
Patterns of substitution of species within and between three distinct broad groups: a) wild marine bony fishes; b) wild marine elasmobranchs; c) freshwater and anadromous bony fishes from aquaculture. Arrows show each of eight observed types of substitutions, and numbers show the observed frequency (%) of each type. Line widths are proportional to the frequency of a given substitution combination observed. The possible substitution combinations are 1) substitution between two marine bony fishes; 2) substitution of an elasmobranch by a marine bony fish; 3) substitution of a marine bony fish by an elasmobranch; 4) substitution of a marine bony fish by a freshwater bony fish from aquaculture 5) substitution between freshwater bony fishes from aquaculture; 6) substitution of a bony fish from aquaculture by a marine bony fish; 7) substitution between elasmobranchs; 8) substitution of an elasmobranch by a freshwater bony fish from aquaculture. The substitution of a freshwater fish from aquaculture by an elasmobranch was the only possible substitution type not observed.

We grouped samples into three main categories depending on their origin: wild marine bony fishes, wild marine elasmobranchs, and freshwater and anadromous bony fishes from aquaculture (Fig 2, S5 Table). Out of 9 possible types of substitutions among these three broad groups (e.g., wild marine bony fish substituted by elasmobranch), we observed them all except the substitution of a freshwater fish from aquaculture by an elasmobranch (Fig 2). From the 116 instances of mislabeling found, the most common, by far (70.6%), involved the substitution between two marine bony fishes (*e*.*g*., *marlin* substituted by yellowfin tuna, roosterfish (*Nematistius pectoralis*) or sailfish (*Istiophorus platypterus*, Figs 1 and 2). The second and third most common substitution types were when a marine bony fish was substituted either by a freshwater fish from aquaculture (17.2%), *e*.*g*., *mero* substituted by swai, or by an elasmobranch (4.3%), *e*.*g*., *marlin* substituted by thresher shark (*Alopias pelagicus*) and silky sharks (*Carcharhinus falciformis*). Other types of substitution showed a relatively low frequency (0.8–2.5%, Fig 2).

### Mislabeling and proxies of net availability and substitutability in our dataset

**Fig 3:**
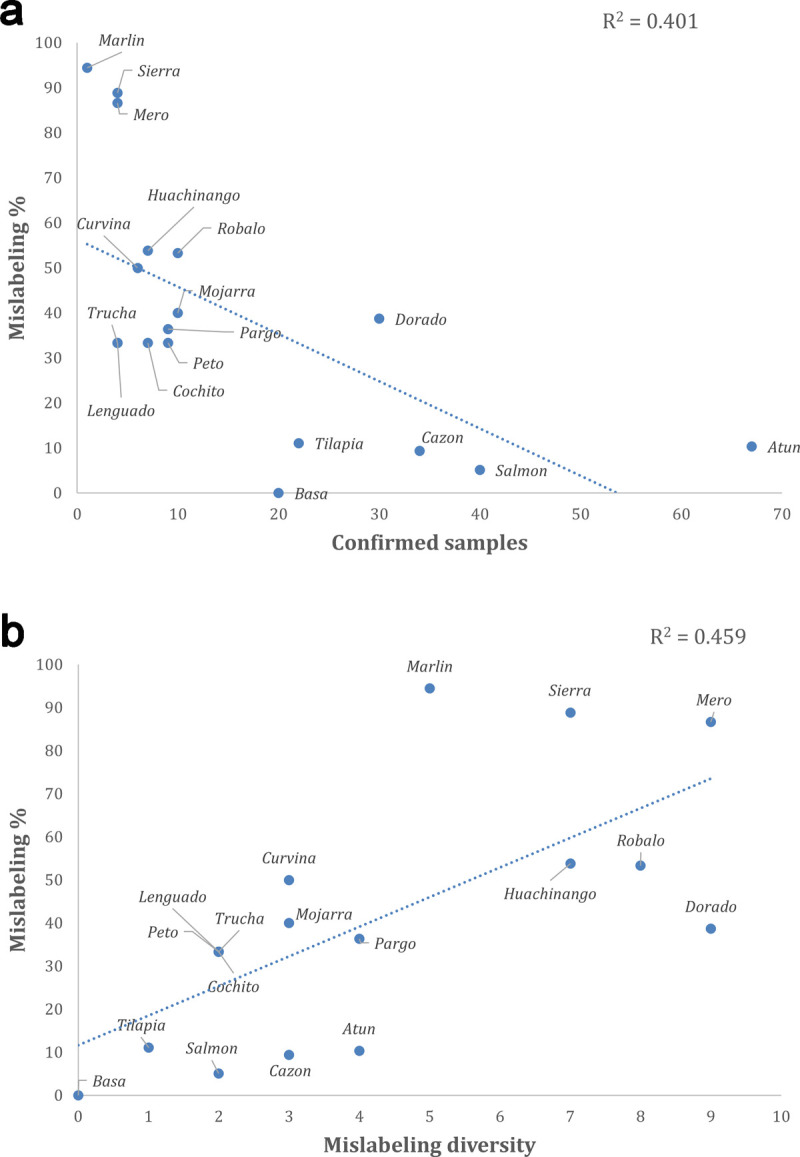
Linear regression analyses showing: **A)** the relationship of net availability of a species in our dataset (Confirmed number of samples, excluding mislabeling and including samples used to substitute other species) as a predictor of mislabeling rates for the 18 most important commercial names found within three cities of Mexico; **B**) the relationship of the number of substitute species sold under the name of the focal species (Mislabeling diversity, a proxy of demand for commercial species in our dataset) as a predictor of mislabeling rates.

Our analyses relating mislabeling rates in commercial names and our measures of net availability and proxies for demand in our dataset based on patterns of substitutability (Table 1) revealed some significant trends:

1. Commercial names associated with species showing low availability in our study (smaller values of confirmed number of samples, observed in commercial names *marlin*, *sierra*, *mero*, *curvina*, *huachinango* and *robalo*) showed significantly higher levels of mislabeling (mislabeling ≥ 53%, Fig 3A, R^2^ = 0.401, *P* = 0.004).
2. From the three proxies of demand–Mislabeling diversity, Substitutability frequency, and Substitutability diversity–obtained from our dataset, we found that commercial names showing a higher number of substitute species (higher Mislabeling diversity) showed significantly higher rates of mislabeling, including *mero*, *dorado*, *robalo*, *huachinango*, *sierra* and *marlin* (Fig 3B, R^2^ = 0.459, P = 0.001).
3. Species that were more frequently used as substitutes for another commercial names (higher Substitutability frequency: *tuna*, *mahi mahi*, *swai* and *tilapia*) showed significantly lower mislabeling rates (R^2^ = 0.225, P = 0.046). In contrast, the number of different species that a given species substituted (Substitutability diversity) was not significantly correlated with mislabeling rates (R^2^ = 0.061, P = 0.320). Importantly, the number of samples analyzed for each commercial name (Verbal sample number in Table 2) was not a significant predictor of mislabeling (R^2^ = 0.147, P = 0.116).

### Net availability from landing and imports data

**Fig 4:**
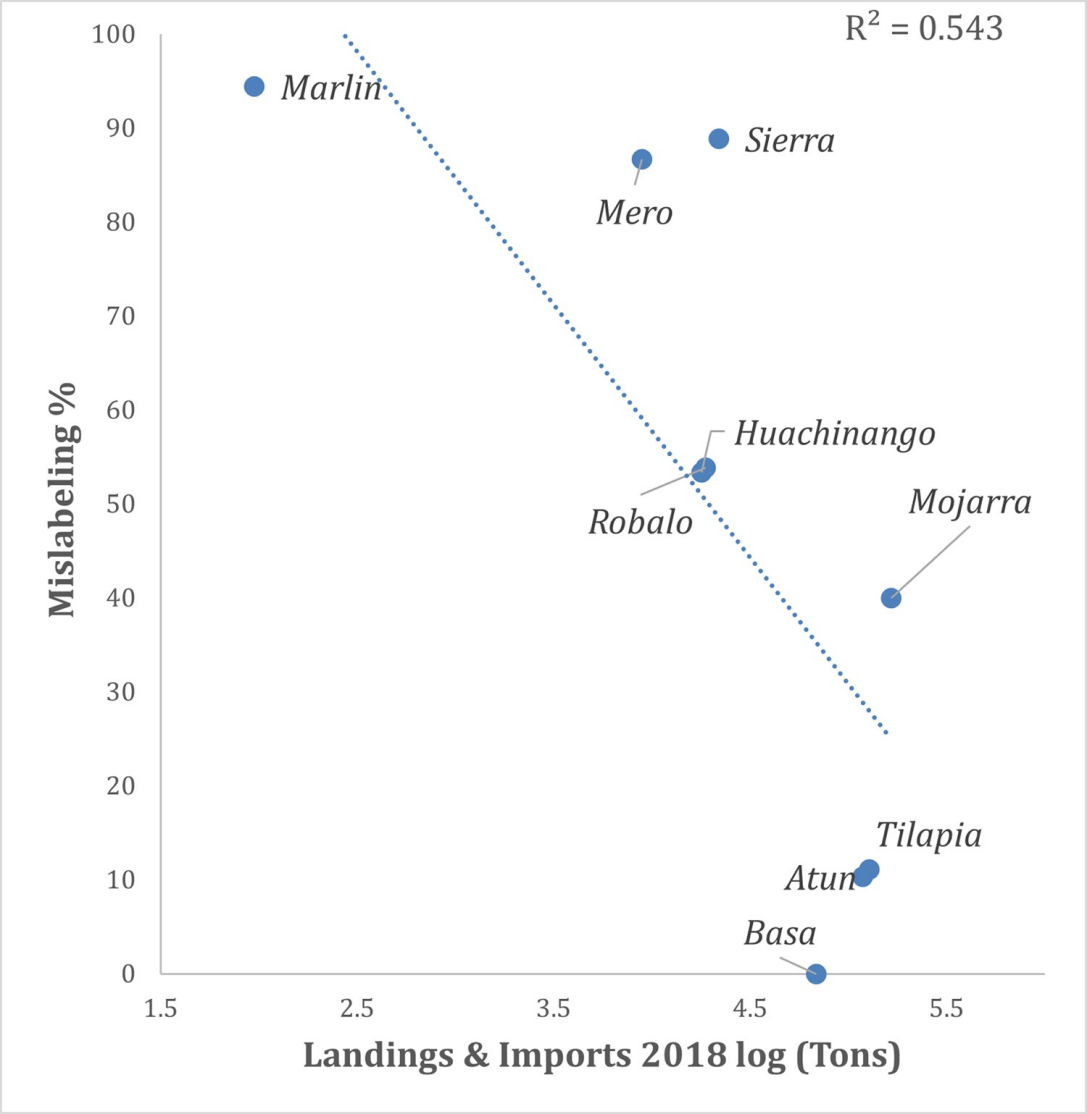
Linear regression analysis shows the relationship between landing and import data for nine of the main commercial names found in Mexico as a predictor of observed mislabeling rates.

Landings and imports for 2018 (Tons per year) are presented in S7 Table for nine of the main commercial species for which official data was available. *Mojarra* was positioned as the species with the largest volume, followed by *tilapia*, *atun* and *basa*. In contrast, the commercial names with the lowest volumes were, in increasing order *marlin*, *mero*, *robalo*, *huachinango* and *sierra*. Landing data (for 2018) significantly explained 54% of the variance observed in mislabeling rates (Fig 4, P = 0.023). Species with higher volumes, including *basa*, *atun* and *tilapia*, showed lower mislabeling rates (≤ 11.1%) than species with lower volumes.

### Threatened species

Among the samples analyzed, we identified 16 threatened (i.e., vulnerable, endangered, or critically endangered) species represented by 26 samples (6.7% of all samples) and four near-threatened species represented by 20 additional samples (5.2%), according to the International Union for the Conservation of Nature’s (IUCN) Red List of Threatened Species (Table 4). These analyses excluded three species that, although considered threatened in their native habitat, are produced in aquaculture operations where our samples more likely originated: silver carp (*Hypophthalmichthys molitrix*), swai (*Pangasianodon hypophthalmus*,) and totoaba (*Totoaba macdonaldi*). Among the threatened species, two were critically endangered (*Anguilla anguilla* and *Sphyrna lewini*), five were endangered *(Anguilla rostrata*, *Lopholatilus chamaeleonticeps*, *Alopias pelagicus*, *Carcharhinus plumbeus*, *Carcharhinus acronotus*) and seven were vulnerable (Table 4). Among the 46 samples identified as threatened and near-threatened species, we observed six instances of mislabeling (13%). Three species of sharks identified are included in Appendix II of the Convention on International Trade in Endangered Species (CITES), including *Sphyrna lewini*, *Alopias pelagicus* and *Carcharhinus falciformis* (Table 4). From these, *A*. *pelagicus* was used as a substitute for *marlin*.

**Table 4:** List of 20 threatened and near threatened species identified in this study, including scientific name, common name, commercial name under which they were sold, if the sample was mislabeled or not, IUCN red list category, and if the species is included in any of CITES appendices, and total number of samples identified.

	Scientific name	Common name	Commercial name	Mislabeled?	IUCN | CITES	Total (numbers of samples identified)
1	*Anguilla anguilla*	European eel	Anguila	No	IUCN Critically endangered	1
2	*Sphyrna lewini*	Scalloped hammerhead shark	Cazón	No	IUCN Critically endangered | CITES Apendix II	1
3	*Anguilla rostrata*	American eel	Anguila	No	IUCN endangered	1
4	*Lopholatilus chamaeleonticeps*	Great northern tilefish	Robalo	Yes	IUCN endangered	1
5	*Alopias pelagicus*	Thresher shark	Marlin	Yes	IUCN endangered | CITES Appendix II	2
6	*Carcharhinus plumbeus*	Sandbar shark	Cazón	No	IUCN endangered	1
7	*Carcharhinus acronotus*	Blacknose shark	Cazón	No	IUCN endangered	1
8	*Thunnus orientalis/Thunnus thynnus*	Pacific/Atlantic bluefin tuna	Atún	No	IUCN Near Threatened /IUCN endangered	2
9	*Carcharhinus falciformis*	Silky shark	Cazón	No	IUCN vulnerable | CITES Appendix II	7
10	*Makaira nigricans/Istiompax indica*	Blue/blackmarlin	Marlin	No	IUCN endangered/Data deficient	1
11	*Lachnolaimus maximus*	Hogfish	Boquinete	No	IUCN vulnerable	2
12	*Hyporthodus acanthistius*	Rooster hind	Robalo	Yes	IUCN vulnerable	1
13	*Lutjanus campechanus*	Red snapper	Huachinango	No	IUCN vulnerable	1
14	*Mycteroperca bonaci*	Black grouper	Mero	No	IUCN vulnerable	1
15	*Carcharhinus leucas*	Bull shark	Cazón	No	IUCN vulnerable	2
16	*Carcharhinus brevipinna*	Spinner shark	Cazón	No	IUCN vulnerable	1
17	*Paralichthys lethostigma*	Southern flounder	Lenguado	No	IUCN Near-threatened	1
18	*Dasyatis americana*	Southern stingray	Mantarraya, huachinango, guitarra	Yes	IUCN Near-threatened	7
19	*Prionace glauca*	Blue shark	Cazón, pescado	No	IUCN Near-threatened	8
20	*Mustelus canis*	Dusky smooth- hound	Cazón	No	IUCN Near-threatened	4
					**Total samples**	**46**

## Discussion

We documented a complex scenario for mislabeling and substitution in the fish trade in three cities within Mexico. While about half of all the samples collected belonged to five species traded globally (yellowfin tuna, Atlantic salmon, mahi mahi, swai and tilapia), the other half were represented by ~100 local species targeted by small-scale fishers. Overall, we found that one of every three samples of fish sold in Mexico was mislabeled, a figure very similar to a previous international assessment of multiple peer-reviewed mislabeling studies [7]. Our results supported the view that patterns of mislabeling and substitution were related to a balance between the availability of a species and its level of demand, where local species in low supply but with high demand are more frequently mislabeled. The species used as substitutes originated from the same two primary sources: the few global species in large supply and a diverse pool of local species from small-scale fisheries. Below we discuss factors affecting availability within each group and implications for improving traceability in Mexico.

The presence of the five most frequent species identified in the samples highlights the importance of the global seafood trade. Except for mahi mahi, they have significant levels of aquaculture or ranching production within Mexico (yellowfin tuna and tilapia) or overseas (yellowfin tuna, Atlantic salmon, swai). These species were commonly used as substitutes for other species, and except again for mahi mahi, showed low mislabeling rates themselves (≤ 11%). Yellowfin tuna was the single most important commodity in terms of available volume, originating from Mexican industrial fisheries landings, national ranching and imports from wild capture and aquaculture [46]. Industrial fisheries for tuna and other large pelagic species have expanded globally over the last six decades, dominated by the Pacific Ocean tuna fisheries for skipjack and yellowfin [48].

Aquaculture seems to increasingly contribute to the dilution effect by providing products that clandestinely substitute some overfished marine capture fishery species [19, 49]. This type of substitution has multiple implications (e.g., larger environmental footprint) beyond those directly affecting the final consumer [50]. The volume of tilapia and swai imported into Mexico approximately doubled from 2011 to 2018 (S7 Table). A worldwide substitution meta-analysis study [9] and a previous mislabeling study in Mexico [37] reported swai as one of the most common substitutes. Atlantic salmon produced in international aquaculture operations is one of the leading imported products in Mexico by volume and value [46].

Our result that nearly 100 local species were sold under 42 different commercial names emphasizes the significant contribution of biological diversity and artisanal fisheries to food security in Mexico. With few exceptions (e.g., silver carp), most of these species are captured by ~200,000 small-scale fishers in Mexico that operate with ~75,000 vessels under 12 m length overall [3]. This critical component of Mexico’s fish trade is sustained by the high levels of marine fish diversity, estimated at ~2,800 known species [51]. Popular local commercial names including *marlin*, *mero*, *robalo*, *mojarra*, *huachinango*, *pargo*, and *sierra*, were consistently over-represented in the market and characterized by the highest mislabeling rates (36.3% to 94.4%). Our analyses of official landing data supported that higher mislabeling was associated with low supply levels for some of these species. Notably, these popular commercial names served to sell a large portion of the 53 species identified as substitutes in our study.

Demand consistently seems to outpace supply in the national market for the most popular commercial species, which are either overfished or fully exploited. In Mexico, fisheries management decisions are made according to the Carta Nacional Pesquera (National Fisheries Chart), which establishes the fisheries status and gives recommendations for each fishery. The grouper fishery (*mero*) has been identified as overfished by the Mexican government [52], and its current abundance is only a third of that calculated in the early 1970s [53]. Local availability is further reduced by the export market for Mexican grouper dominated by the US [54]. The snapper fishery (*pargo*) is officially recognized as deteriorated or at its Maximum Sustainable Yield in the Gulf of Mexico [52]. It is also one of the most widely mislabeled species in the US [55] and one of the most popular and controversial fisheries in the South Atlantic and Gulf of Mexico [56]. Snook (*robalo*) is officially at its Maximum Sustainable Yield for the Gulf of Mexico and the Caribbean Sea, and a monitoring plan was recommended to be implemented for its assessment [52]. Sierra and red snapper (*huachinango*) fisheries lack specific management tools–a National Official Norm and a Fisheries Management Plan–thus being considered fisheries with less effective management. The most extreme case from the popular but highly mislabeled local species group was marlin, which was frequently substituted by yellowfin tuna. Only 95 tons of marlin are officially allowed to be caught annually exclusively by sport fisheries and as bycatch [57], while recreational catches of white marlin *(Tetrapturus spp*.) have declined since the late 70s [58].

Two commercial names that deserve further studies are mahi mahi (*dorado*) and shark meat (*cazon*). Mahi mahi (*dorado*) seems to be an exception in its mixed patterns of relatively high mislabeling (39%) while also frequently used as a substitute for ten commercial names. This pattern suggests that high mislabeling of mahi-mahi is not driven by low availability and that other unknown factors are at play to explain its prevalence in the market as a concealed substitute species, a pattern previously reported in the Peruvian seafood sector [23]. Like marlin, mahi mahi is legally restricted to recreational fisheries in Mexico (only for self-consumption), and a minimum percentage, from 4 to 10%, to bycatch for finfish and shark fisheries which can be commercialized [59, 60].

*Cazon* is a generic name with ambiguous meaning in Mexico. Sometimes *cazon* is understood by fisheries authorities to mean smaller sharks like Atlantic sharpnose shark (*Rhizoprionodon terraenovae*), but is also often used by those in the seafood trade to mean any shark meat. Although less than 10% of *cazon* samples collected were mislabeled, we found that using a generic name allows the entry to the market of IUCN threatened species that are subject to international trade restrictions under CITES, concealing their real identity from regulators and consumers. For example, we documented the use of *cazon* as a commercial umbrella name for IUCN critically endangered species like scalloped hammerhead shark (*Sphyrna lewini*), endangered sandbar shark (*Carcharhinus plumbeus)* and blacknose shark (*C*. *acronotus*) and vulnerable silky shark (*C*. *falciformis*), bull shark (*C*. *leucas*) and spinner shark (*C*. *brevipinna*). We found 13 shark species sold as *cazon*, mainly represented by the IUCN near-threatened blue shark (*Prionace glauca)* and Dusky smooth-hound shark (*Mustelus canis*), IUCN vulnerable silky shark and IUCN least concern Atlantic sharpnose shark. A previous mislabeling study in Mexico [37] also identified several species listed as threatened in the IUNC Red List. The shark fishery in Mexico is reported to be at its Maximum Sustainable Yield level [52]. It would be useful to conduct a more comprehensive sampling of seafood products labeled *cazon* to identify all the species sold under this commercial name, dismiss the *cazon* label, and employ a specific commercial name for each shark species.

### Implications for traceability in Mexico

We found that purchasing a fish sample with a written label indicating the commercial name associated with it (either in a grocery store or in a restaurant menu) decreased the chances of mislabeling compared to when a commercial name was mentioned verbally. This effect was particularly evident in grocery stores and can be explained by supermarket chains having more robust controls over their product provision than other retailers [61]. A recent study found that species labeling through the Marine Stewardship Council certification scheme reduced mislabeling below 1% among 27 species across 18 countries [62]. Thus, a minimum traceability standard requiring a written indication of the commercial name being sold might help reduce mislabeling.

Understanding the scope, scale, and trends of seafood mislabeling is essential for consumers, fisheries managers, and participants in the seafood supply chain. Whether intentional or unintentional, Seafood fraud weakens public trust, compromises consumers’ ability to adhere to dietary restrictions, and poses public health concerns [63, 64]. A mandatory traceability system along the entire value chain represents a key solution for consumers, the economy surrounding these fisheries, and even better fisheries management. One of the main components of such a traceability scheme is clear product labeling. The European Regulation [65] on the common organization of the markets in fishery and aquaculture products indicates that mandatory information must be displayed on fish labels (the trade name of the species and its scientific name, the production method, the area where the product was caught or farmed). Additionally, the Member States must publish a list of the trade names accepted in their territory and the corresponding scientific names. Beginning with an official equivalency table that allows commercial names identification throughout Mexico and its scientific equivalent is a starting point proposed for this traceability system.

An essential outcome of a traceability system is increasing consumer trust in aquaculture and wild-caught products. Consumers often have concerns about the safety and sustainability of some aquaculture products, which could promote the implementation of quality management systems addressing the need for transparent information along the entire food chain, supported by modern traceability methods [66]. In Mexico, traceability may help promote trust among consumers related to the food safety of the growing national aquaculture industry. Although DNA barcoding procedures for identifying mislabeling may be costly and relatively time-consuming, technological breakthroughs are frequently introduced, and they may be used to streamline sample collection and identification that could support a successful traceability system [67]. New methodologies in development could help to genetically identify species originating from diverse regions and countries [68].

In Mexico, it is crucial to have a traceability policy that includes elements that allow verifiable data for the appropriate authorities to determine the product’s legal origin, safety, quality, and veracity. All these aspects are vital pieces for consumer decision-making. Some strategic points for implementing a traceability system along the supply chain including shipboard, landing sites (harbor), collection centers, storage plants, processing plants, export plants, transport and final points of sale. It is also important that official sources standardize data formats to enable comparative studies of seafood products nationwide. Currently, mislabeling occurs in Mexico in the absence of specific legislation and transparent rules for implementing basic labeling standards.

## Supporting information

S1 Table List of the 18 main commercial names in Spanish and the species associated to them when commercialized. (XLSX)

S2 Table Raw data including detailed taxonomic identifications for each of the samples analyzed according to BOLD and GenBank databases. (XLSX)

S3 Table List and frequency of 48 commercial names under which 376 samples were sold. (DOCX)

S4 Table List and frequency of 103 species identified via genetic barcoding of COI gene. (DOCX)

S5 Table List of 90 unique combinations for 116 instances of mislabeling observed, including 53 species used as a substitute and 32 commercial names they substituted, and the frequency observed. (DOCX)

S6 Table Mislabeling rates for commercial names in three cities of Mexico. (DOCX)

S7 Table 2010–2018 official landing and imports data in tons for nine of the main commercial species identified. (XLSX)
