# Supplementary material for: DNA barcoding reveals global and local influences on patterns of mislabeling and substitution in the trade of fish in Mexico
Source: PLoS One. 2022 Apr 14;17(4):e0265960. doi: 10.1371/journal.pone.0265960 (PMC9009668; doi:10.1371/journal.pone.0265960)
Supplement: S3 Table — (DOCX) [file pone.0265960.s003.docx]

**S3 Table.** List of 48 commercial names under which 376 samples were sold, showing their presence in 1-3 cities within Mexico in decreasing order according to their observed sample size (N).

| **No.** | **Commercial name** | **Number of cities on which it was present** | **N** |
| --- | --- | --- | --- |
| 1 | Atún | 3 | 58 |
| 2 | Salmón | 3 | 39 |
| 3 | Cazón | 3 | 32 |
| 4 | Dorado | 3 | 31 |
| 5 | Marlin | 3 | 18 |
| 6 | Tilapia | 3 | 18 |
| 7 | Mero | 2 | 15 |
| 8 | Robalo | 2 | 15 |
| 9 | Mojarra | 3 | 15 |
| 10 | Huachinango | 3 | 13 |
| 11 | Basa | 3 | 12 |
| 12 | Pargo | 2 | 11 |
| 13 | Sierra | 2 | 9 |
| 14 | Curvina | 2 | 6 |
| 15 | Cochito | 1 | 6 |
| 16 | Lenguado | 3 | 6 |
| 17 | Peto | 1 | 6 |
| 18 | Trucha | 1 | 6 |
| 19 | Mantarraya | 3 | 6 |
| 20 | Merluza | 3 | 5 |
| 21 | Pez espada | 1 | 5 |
| 22 | Esmedregal | 2 | 4 |
| 23 | Hamachi | 3 | 3 |
| 24 | Bacalao | 1 | 3 |
| 25 | Botete | 1 | 3 |
| 26 | Pez vela | 2 | 3 |
| 27 | Abadejo | 1 | 2 |
| 28 | Anguila | 2 | 2 |
| 29 | Boquinete | 1 | 2 |
| 30 | Lisa | 2 | 2 |
| 31 | Coronado | 1 | 2 |
| 32 | Jurel | 1 | 2 |
| 33 | Anchoa del Cantábrico | 1 | 1 |
| 34 | Black Cod | 1 | 1 |
| 35 | Garropa | 1 | 1 |
| 36 | Pajarito | 1 | 1 |
| 37 | Pámpano | 1 | 1 |
| 38 | Totoaba | 1 | 1 |
| 39 | Xcochin | 1 | 1 |
| 40 | Blanco de oriente | 1 | 1 |
| 41 | Charal | 1 | 1 |
| 42 | Lobina | 1 | 1 |
| 43 | Pescado blanco | 1 | 1 |
| 44 | Pez bobo | 1 | 1 |
| 45 | Pez volador | 1 | 1 |
| 46 | Sibas | 1 | 1 |
| 47 | Tiburón azul | 1 | 1 |
| 48 | Tiburón guitarra | 1 | 1 |
|  | **Total** |  | **376** |
