# Supplementary material for: DNA barcoding reveals global and local influences on patterns of mislabeling and substitution in the trade of fish in Mexico
Source: PLoS One. 2022 Apr 14;17(4):e0265960. doi: 10.1371/journal.pone.0265960 (PMC9009668; doi:10.1371/journal.pone.0265960)
Supplement: S4 Table — (DOCX) [file pone.0265960.s004.docx]

**S4 Table.** List of 103 species identified via genetic barcoding of COI gene, showing sample size (N), relative frequency (%) and cumulative frequency. Species ordered in decreasing relative frequency.

| **No.** | **Species** | **N** | **%** | **Cumulative**  **%** |
| --- | --- | --- | --- | --- |
| 1 | *Thunnus albacares* | 64 | 16.7 | 16.7 |
| 2 | *Salmo salar* | 33 | 8.6 | 25.3 |
| 3 | *Coryphaena hippurus* | 30 | 7.8 | 33.2 |
| 4 | *Oreochromis niloticus* | 24 | 6.3 | 39.4 |
| 5 | *Pangasianodon hypophthalmus* | 23 | 6.0 | 45.4 |
| 6 | *Centropomus undecimalis* | 8 | 2.1 | 47.5 |
| 7 | *Prionace glauca* | 8 | 2.1 | 49.6 |
| 8 | *Xiphias gladius* | 8 | 2.1 | 51.7 |
| 9 | *Carcharhinus falciformis* | 7 | 1.8 | 53.5 |
| 10 | *Dasyatis americana* | 7 | 1.8 | 55.4 |
| 11 | *Scomberomorus cavalla* | 6 | 1.6 | 56.9 |
| 12 | *Seriola rivoliana* | 6 | 1.6 | 58.5 |
| 13 | *Balistes polylepis* | 5 | 1.3 | 59.8 |
| 14 | *Oncorhynchus gorbuscha* | 5 | 1.3 | 61.1 |
| 15 | *Pagrus pagrus* | 5 | 1.3 | 62.4 |
| 16 | *Rhizoprionodon terraenovae* | 5 | 1.3 | 63.7 |
| 17 | *Hypophthalmichthys molitrix* | 4 | 1.0 | 64.8 |
| 18 | *Mustelus canis* | 4 | 1.0 | 65.8 |
| 19 | *Oncorhynchus mykiss* | 4 | 1.0 | 66.8 |
| 20 | *Acanthocybium solandri* | 3 | 0.8 | 67.6 |
| 21 | *Anoplopoma fimbria* | 3 | 0.8 | 68.4 |
| 22 | *Ctenopharyngodon idella* | 3 | 0.8 | 69.2 |
| 23 | *Lutjanus colorado* | 3 | 0.8 | 70.0 |
| 24 | *Lutjanus guttatus* | 3 | 0.8 | 70.8 |
| 25 | *Merluccius productus* | 3 | 0.8 | 71.5 |
| 26 | *Merluccius productus/Merluccius angustimanus* | 3 | 0.8 | 72.3 |
| 27 | *Oreochromis aureus* | 3 | 0.8 | 73.1 |
| 28 | *Peprilus snyderi/Peprilus paru* | 3 | 0.8 | 73.9 |
| 29 | *Seriola dumerili* | 3 | 0.8 | 74.7 |
| 30 | *Seriola quinqueradiata* | 3 | 0.8 | 75.5 |
| 31 | *Alopias pelagicus* | 2 | 0.5 | 76.0 |
| 32 | *Bagre marinus* | 2 | 0.5 | 76.5 |
| 33 | *Balistes vetula* | 2 | 0.5 | 77.0 |
| 34 | *Brotula clarkae* | 2 | 0.5 | 77.5 |
| 35 | *Carcharhinus leucas* | 2 | 0.5 | 78.1 |
| 36 | *Centropomus viridis* | 2 | 0.5 | 78.6 |
| 37 | *Cynoscion reticulatus* | 2 | 0.5 | 79.1 |
| 38 | *Cynoscion xanthulus* | 2 | 0.5 | 79.6 |
| 39 | *Gadus chalcogrammus* | 2 | 0.5 | 80.2 |
| 40 | *Hyporthodus flavolimbatus* | 2 | 0.5 | 80.7 |
| 41 | *Istiophorus platypterus* | 2 | 0.5 | 81.2 |
| 42 | *Lachnolaimus maximus* | 2 | 0.5 | 81.7 |
| 43 | *Lutjanus griseus* | 2 | 0.5 | 82.2 |
| 44 | *Mugil cephalus* | 2 | 0.5 | 82.8 |
| 45 | *Oncorhynchus keta* | 2 | 0.5 | 83.3 |
| 46 | *Opisthonema libertate* | 2 | 0.5 | 83.8 |
| 47 | *Oreochromis niloticus/Oreochromis mossambicus* | 2 | 0.5 | 84.3 |
| 48 | *Rachycentron canadum* | 2 | 0.5 | 84.9 |
| 49 | *Scomberomorus sierra* | 2 | 0.5 | 85.4 |
| 50 | *Sphyraena barracuda* | 2 | 0.5 | 85.9 |
| 51 | *Thunnus orientalis/Thunnus thynnus* | 2 | 0.5 | 86.4 |
| 52 | *Anchoa lyolepis* | 1 | 0.3 | 86.7 |
| 53 | *Anguilla anguilla* | 1 | 0.3 | 86.9 |
| 54 | *Anguilla rostrata* | 1 | 0.3 | 87.2 |
| 55 | *Bodianus diplotaenia* | 1 | 0.3 | 87.5 |
| 56 | *Carcharhinus acronotus* | 1 | 0.3 | 87.7 |
| 57 | *Carcharhinus brevipinna* | 1 | 0.3 | 88.0 |
| 58 | *Carcharhinus plumbeus* | 1 | 0.3 | 88.3 |
| 59 | *Caulolatilus microps* | 1 | 0.3 | 88.5 |
| 60 | *Cephalopholis fulva* | 1 | 0.3 | 88.8 |
| 61 | *Chanos chanos* | 1 | 0.3 | 89.0 |
| 62 | *Chloroscombrus chrysurus* | 1 | 0.3 | 89.3 |
| 63 | *Cynoscion albus* | 1 | 0.3 | 89.6 |
| 64 | *Cynoscion parvipinnis* | 1 | 0.3 | 89.8 |
| 65 | *Dasyatis centroura/Dasyatis guttata* | 1 | 0.3 | 90.1 |
| 66 | *Diapterus brevirostris* | 1 | 0.3 | 90.3 |
| 67 | *Engraulis encrasicolus* | 1 | 0.3 | 90.6 |
| 68 | *Epinephelus guttatus* | 1 | 0.3 | 90.9 |
| 69 | *Gadus macrocephalus* | 1 | 0.3 | 91.1 |
| 70 | *Hemiramphus saltator* | 1 | 0.3 | 91.4 |
| 71 | *Hyporthodus acanthistius* | 1 | 0.3 | 91.6 |
| 72 | *Lobotes pacificus* | 1 | 0.3 | 91.9 |
| 73 | *Lopholatilus chamaeleonticeps* | 1 | 0.3 | 92.2 |
| 74 | *Lutjanus analis* | 1 | 0.3 | 92.4 |
| 75 | *Lutjanus campechanus* | 1 | 0.3 | 92.7 |
| 76 | *Lutjanus novemfasciatus* | 1 | 0.3 | 93.0 |
| 77 | *Lutjanus purpureus/Lutjanus campechanus* | 1 | 0.3 | 93.2 |
| 78 | *Lutjanus vivanus* | 1 | 0.3 | 93.5 |
| 79 | *Makaira nigricans/Istiompax indica* | 1 | 0.3 | 93.7 |
| 80 | *Mallotus villosus* | 1 | 0.3 | 94.0 |
| 81 | *Merluccius angustimanus* | 1 | 0.3 | 94.3 |
| 82 | *Merluccius australis* | 1 | 0.3 | 94.5 |
| 83 | *Mugil curema* | 1 | 0.3 | 94.8 |
| 84 | *Mustelus californicus* | 1 | 0.3 | 95.0 |
| 85 | *Mustelus henlei* | 1 | 0.3 | 95.3 |
| 86 | *Mycteroperca bonaci* | 1 | 0.3 | 95.6 |
| 87 | *Nematistius pectoralis* | 1 | 0.3 | 95.8 |
| 88 | *Oreochromis aureus/Oreochromis niloticus* | 1 | 0.3 | 96.1 |
| 89 | *Oreochromis mossambicus* | 1 | 0.3 | 96.3 |
| 90 | *Orthopristis chalceus* | 1 | 0.3 | 96.6 |
| 91 | *Paralichthys californicus* | 1 | 0.3 | 96.9 |
| 92 | *Paralichthys lethostigma* | 1 | 0.3 | 97.1 |
| 93 | *Paranthias colonus* | 1 | 0.3 | 97.4 |
| 94 | *Pleuronectes platessa* | 1 | 0.3 | 97.7 |
| 95 | *Sciades seemanni* | 1 | 0.3 | 97.9 |
| 96 | *Sphoeroides annulatus* | 1 | 0.3 | 98.2 |
| 97 | *Sphoeroides lispus* | 1 | 0.3 | 98.4 |
| 98 | *Sphyraena ensis* | 1 | 0.3 | 98.7 |
| 99 | *Sphyrna lewini* | 1 | 0.3 | 99.0 |
| 100 | *Thunnus albacares/Thunnus tonggol* | 1 | 0.3 | 99.2 |
| 101 | *Totoaba macdonaldi* | 1 | 0.3 | 99.5 |
| 102 | *Trachinotus carolinus* | 1 | 0.3 | 99.7 |
| 103 | *Xystreurys liolepis* | 1 | 0.3 | 100.0 |
|  | **Total** | **383** |  | **100.0** |
