# Supplementary material for: DNA barcoding reveals global and local influences on patterns of mislabeling and substitution in the trade of fish in Mexico
Source: PLoS One. 2022 Apr 14;17(4):e0265960. doi: 10.1371/journal.pone.0265960 (PMC9009668; doi:10.1371/journal.pone.0265960)
Supplement: S5 Table — (DOCX) [file pone.0265960.s005.docx]

**S5 Table**. List of 90 unique combinations for 116 instances of mislabeling observed, including 53 species used as substitute and 32 commercial names they substituted, and the frequency observed. We also show the type of substitution of species within and between three distinct groups: a) wild marine bony fishes; b) wild marine elasmobranchs; c) freshwater and anadromous bony fishes from aquaculture. Nine possible types of substitutions, include: 1) substitution between two marine bony fishes; 2) substitution of an elasmobranch by a marine bony fish; 3) substitution of a marine bony fish by an elasmobranch; 4) substitution of a marine bony fish by a freshwater bony fish from aquaculture 5) substitution between freshwater bony fishes from aquaculture; 6) substitution of a bony fish from aquaculture by a marine bony fish; 7) substitution between elasmobranchs; 8) substitution of an elasmobranch by a freshwater bony fish from aquaculture; 9) substitution of a freshwater bony fish of aquaculture by an elasmobranch.

| **Species genetically identified -**  **Common name** | **Commercial name** | **Frequency** | **Substitution**  **type** |
| --- | --- | --- | --- |
| *Acanthocybium solandri*-Wahoo | Dorado | 3 | 1 |
| *Alopias pelagicus*-Pelagic thresher shark | Marlin | 2 | 3 |
| *Anchoa lyolepis*-Dusky anchovy | Charal | 1 | 1 |
| *Anoplopoma fimbria*-Sablefish | Dorado | 1 | 1 |
| *Bagre marinus*-Gafftopsail Catfish | Huachinango | 1 | 1 |
| *Bagre marinus*-Gafftopsail Catfish | Mero | 1 | 1 |
| *Balistes polylepis*-Finescale triggerfish | Atun | 2 | 1 |
| *Balistes vetula*-Cochito reina | Mojarra | 1 | 1 |
| *Bodianus diplotaenia*-Mexican hogfish | Pargo | 1 | 1 |
| *Brotula clarkae*-Pacific Bearded Brotula | Huachinango | 1 | 1 |
| *Brotula clarkae*-Pacific Bearded Brotula | Lenguado | 1 | 1 |
| *Carcharhinus falciformis*-Silky Shark | Marlin | 1 | 3 |
| *Carcharhinus falciformis*-Silky Shark | Tiburon Azul | 1 | 7 |
| *Caulolatilus microps*-Blueline Tilefish | Mero | 1 | 1 |
| *Centropomus undecimalis*-Common Snook | Atun | 1 | 1 |
| *Centropomus undecimalis*-Common Snook | Mero | 1 | 1 |
| *Centropomus viridis*-White Snook | Pargo | 1 | 1 |
| *Cephalopholis fulva*-Coney | Mero | 1 | 1 |
| *Chanos chanos*-Milkfish | Sierra | 1 | 1 |
| *Coryphaena hippurus*-Mahi mahi | Atun | 2 | 1 |
| *Coryphaena hippurus* -Mahi mahi | Botete | 1 | 1 |
| *Coryphaena hippurus*-Mahi mahi | Cochito | 1 | 1 |
| *Coryphaena hippurus*-Mahi mahi | Esmedregal | 1 | 1 |
| *Coryphaena hippurus*-Mahi mahi | Huachinango | 1 | 1 |
| *Coryphaena hippurus*-Mahi mahi | Lenguado | 1 | 1 |
| *Coryphaena hippurus*-Mahi mahi | Mojarra | 1 | 1 |
| *Coryphaena hippurus*-Mahi mahi | Pez Vela | 1 | 1 |
| *Coryphaena hippurus*-Mahi mahi | Salmon | 1 | 1 |
| *Coryphaena hippurus*-Mahi mahi | Sea bass | 1 | 1 |
| *Ctenopharyngodon idella*-Grass Carp | Cazon | 1 | 8 |
| *Ctenopharyngodon idella*-Grass Carp | Mero | 1 | 4 |
| *Ctenopharyngodon idella*-Grass Carp | Pez bobo | 1 | 4 |
| *Cynoscion reticulatus*-Striped Corvina | Robalo | 1 | 1 |
| *Cynoscion xanthulus*-Orangemouth corvina | Pargo | 1 | 1 |
| *Cynoscion xanthulus*-Orangemouth corvina | Robalo | 1 | 1 |
| *Dasyatis americana*-Southern Stingray | Huachinango | 1 | 3 |
| *Dasyatis americana*-Southern Stingray | Tiburon Guitarra | 1 | 7 |
| *Diapterus brevirostris*-Peruvian mojarra | Sierra | 1 | 1 |
| *Hypophthalmichthys molitrix*-Silver Carp | Bacalao | 1 | 4 |
| *Hypophthalmichthys molitrix*-Silver Carp | Peto | 1 | 4 |
| *Hypophthalmichthys molitrix*-Silver Carp | Robalo | 1 | 4 |
| *Hypophthalmichthys molitrix*-Silver Carp | Sierra | 1 | 4 |
| *Hyporthodus acanthistius*-Gulf Coney | Robalo | 1 | 1 |
| *Hyporthodus flavolimbatus*-Yellowedge Grouper | Huachinango | 1 | 1 |
| *Istiophorus platypterus*-Sailfish | Cazon | 1 | 2 |
| *Istiophorus platypterus*-Sailfish | Marlin | 1 | 1 |
| *Lobotes pacificus*-West Coast Tripletail | Pargo | 1 | 1 |
| *Lopholatilus chamaeleonticeps*-Blue Tilefish | Robalo | 1 | 1 |
| *Lutjanus colorado*-Colorado Snapper | Robalo | 1 | 1 |
| *Mallotus villosus*-Capelin | Pez Volador | 1 | 1 |
| *Merluccius productus*-Pacific Whiting | Dorado | 1 | 1 |
| *Mugil curema*-White Mullet | Sierra | 1 | 1 |
| *Mustelus californicus*-Grey Smoothhound | Jurel | 1 | 3 |
| *Nematistius pectoralis*-Roosterfish | Marlin | 1 | 1 |
| *Opisthonema libertate*-Deepbody Thread Herring | Mero | 1 | 1 |
| *Oreochromis aureus*-Blue Tilapia | Dorado | 1 | 4 |
| *Oreochromis niloticus*-Nile Tilapia | Blanco de Oriente | 1 | 4 |
| *Oreochromis niloticus*-Nile Tilapia | Curvina | 1 | 4 |
| *Oreochromis niloticus*-Nile Tilapia | Mero | 1 | 4 |
| *Oreochromis niloticus*-Nile Tilapia | Robalo | 1 | 4 |
| *Oreochromis niloticus*-Nile Tilapia | Trucha | 1 | 5 |
| *Orthopristis chalceus*-Humpback grunt | Sierra | 1 | 1 |
| *Pagrus pagrus*-Red Porgy | Mojarra | 4 | 1 |
| *Pangasianodon hypophthalmus*-Swai | Mero | 5 | 4 |
| *Pangasianodon hypophthalmus*-Swai | Pescado Blanco | 1 | 4 |
| *Pangasianodon hypophthalmus*-Swai | Tilapia | 2 | 5 |
| *Paranthias colonus*-Pacific Creolefish | Huachinango | 1 | 1 |
| *Peprilus snyderi*-Salema butterfish | Dorado | 1 | 1 |
| *Peprilus snyderi*-Salema butterfish | Sierra | 2 | 1 |
| *Rachycentron canadum*-Cobia | Robalo | 1 | 1 |
| *Salmo salar*-Atlantic Salmon | Atun | 2 | 4 |
| *Salmo salar*-Atlantic Salmon | Trucha | 1 | 5 |
| *Sciades seemanni*-Tete sea catfish | Curvina | 1 | 1 |
| *Scomberomorus cavalla*-King Mackerel | Lobina | 1 | 6 |
| *Scomberomorus cavalla*-King Mackerel | Sierra | 1 | 1 |
| *Scomberomorus sierra*-Pacific Sierra | Cazon | 1 | 2 |
| *Seriola dumerili*-Greater Amberjack | Dorado | 1 | 1 |
| *Seriola rivoliana*-Almaco Jack | Dorado | 1 | 1 |
| *Seriola rivoliana*-Almaco Jack | Huachinango | 1 | 1 |
| *Seriola rivoliana*-Almaco Jack | Mero | 1 | 1 |
| *Seriola rivoliana*-Almaco Jack | Peto | 1 | 1 |
| *Sphyraena barracuda*-Great Barracuda | Coronado | 1 | 1 |
| *Sphyraena barracuda*-Great Barracuda | Dorado | 1 | 1 |
| *Sphyraena ensis*-Vicuda | Curvina | 1 | 1 |
| *Thunnus albacares*-Yellowfin Tuna | Cochito | 1 | 1 |
| *Thunnus albacares*-Yellowfin Tuna | Marlin | 12 | 1 |
| *Thunnus albacares*-Yellowfin Tuna | Pez Vela | 1 | 1 |
| *Thunnus albacares*-Yellowfin Tuna | Salmon | 1 | 6 |
| *Xiphias gladius*-Swordfish | Dorado | 2 | 1 |
| *Xiphias gladius*-Swordfish | Pez Vela | 1 | 1 |
