# Supplementary material for: DNA barcoding reveals global and local influences on patterns of mislabeling and substitution in the trade of fish in Mexico
Source: PLoS One. 2022 Apr 14;17(4):e0265960. doi: 10.1371/journal.pone.0265960 (PMC9009668; doi:10.1371/journal.pone.0265960)
Supplement: S6 Table — (DOCX) [file pone.0265960.s006.docx]

**S6 Table.** Mislabeling rates for commercial names in three cities of Mexico, including 23 commercial names found in Mazatlan, 37 commercial names found in Mexico City, and 23 commercial names found in Cancun. We show commercial name, sample size (N), observed frequency of mislabeling and mislabeling rate (%).

|  | **Mazatlan** |  |  |  |
| --- | --- | --- | --- | --- |
| **No.** | **Commercial name** | **N** | **Mislabeling Frequency** | **Mislabeling rate %** |
| 1 | Atún | 21 | 3 | 14.3 |
| 2 | Dorado | 19 | 3 | 15.8 |
| 3 | Salmón | 10 | 1 | 10.0 |
| 4 | Cazón | 8 | 2 | 25.0 |
| 5 | Marlin | 8 | 8 | 100.0 |
| 6 | Pargo | 8 | 4 | 50.0 |
| 7 | Cochito | 6 | 2 | 33.3 |
| 8 | Sierra | 6 | 6 | 100.0 |
| 9 | Curvina | 5 | 2 | 40.0 |
| 10 | Pez espada | 5 | 0 | 0.0 |
| 11 | Tilapia | 5 | 0 | 0.0 |
| 12 | Botete | 3 | 1 | 33.3 |
| 13 | Robalo | 3 | 2 | 66.7 |
| 14 | Basa | 2 | 0 | 0.0 |
| 15 | Huachinango | 2 | 0 | 0.0 |
| 16 | Lenguado | 2 | 1 | 50.0 |
| 17 | Hamachi | 1 | 0 | 0.0 |
| 18 | Mantarraya | 1 | 0 | 0.0 |
| 19 | Merluza | 1 | 0 | 0.0 |
| 20 | Mojarra | 1 | 0 | 0.0 |
| 21 | Pajarito | 1 | 0 | 0.0 |
| 22 | Pez vela | 1 | 1 | 100.0 |
| 23 | Sibas | 1 | 1 | 100.0 |
|  | **Total** | **120** | **37** | **30.8** |

|  | **Mexico City** |  |  |  |
| --- | --- | --- | --- | --- |
| **No.** | **Commercial name** | **N** | **Mislabeling Frequency** | **Mislabeling**  **rate %** |
| 1 | Atún | 19 | 2 | 10.5 |
| 2 | Robalo | 12 | 6 | 50.0 |
| 3 | Salmón | 12 | 0 | 0.0 |
| 4 | Cazón | 9 | 0 | 0.0 |
| 5 | Huachinango | 9 | 7 | 77.8 |
| 6 | Mojarra | 9 | 1 | 11.1 |
| 7 | Dorado | 7 | 6 | 85.7 |
| 8 | Marlin | 6 | 5 | 83.3 |
| 9 | Peto | 6 | 2 | 33.3 |
| 10 | Trucha | 6 | 2 | 33.3 |
| 11 | Basa | 5 | 0 | 0.0 |
| 12 | Mero | 5 | 5 | 100.0 |
| 13 | Tilapia | 5 | 0 | 0.0 |
| 14 | Mantarraya | 4 | 0 | 0.0 |
| 15 | Bacalao | 3 | 1 | 33.3 |
| 16 | Esmedregal | 3 | 1 | 33.3 |
| 17 | Lenguado | 3 | 1 | 33.3 |
| 18 | Merluza | 3 | 0 | 0.0 |
| 19 | Sierra | 3 | 2 | 66.7 |
| 20 | Abadejo | 2 | 0 | 0.0 |
| 21 | Jurel | 2 | 1 | 50.0 |
| 22 | Pez vela | 2 | 2 | 100.0 |
| 23 | Anchoa del Cantábrico | 1 | 0 | 0.0 |
| 24 | Anguila | 1 | 0 | 0.0 |
| 25 | Black Cod | 1 | 0 | 0.0 |
| 26 | Blanco de oriente | 1 | 1 | 100.0 |
| 27 | Charal | 1 | 1 | 100.0 |
| 28 | Garropa | 1 | 0 | 0.0 |
| 29 | Hamachi | 1 | 0 | 0.0 |
| 30 | Lisa | 1 | 0 | 0.0 |
| 31 | Lobina | 1 | 1 | 100.0 |
| 32 | Pámpano | 1 | 0 | 0.0 |
| 33 | Pez bobo | 1 | 1 | 100.0 |
| 34 | Pez volador | 1 | 1 | 100.0 |
| 35 | Tiburón azul | 1 | 1 | 100.0 |
| 36 | Tiburón guitarra | 1 | 1 | 100.0 |
| 37 | Totoaba | 1 | 0 | 0.0 |
|  | **Total** | **150** | **51** | **34.0** |

|  | **Cancun** |  |  |  |
| --- | --- | --- | --- | --- |
| **No.** | **Commercial name** | **N** | **Mislabeling Frequency** | **Mislabeling rate %** |
| 1 | Atún | 18 | 1 | 5.6 |
| 2 | Salmón | 17 | 1 | 5.9 |
| 3 | Cazón | 15 | 1 | 6.7 |
| 4 | Mero | 10 | 8 | 80.0 |
| 5 | Tilapia | 8 | 2 | 25.0 |
| 6 | Basa | 5 | 0 | 0.0 |
| 7 | Dorado | 5 | 3 | 60.0 |
| 8 | Mojarra | 5 | 5 | 100.0 |
| 9 | Marlin | 4 | 4 | 100.0 |
| 10 | Pargo | 3 | 0 | 0.0 |
| 11 | Boquinete | 2 | 0 | 0.0 |
| 12 | Coronado | 2 | 1 | 50.0 |
| 13 | Huachinango | 2 | 0 | 0.0 |
| 14 | Anguila | 1 | 0 | 0.0 |
| 15 | Curvina | 1 | 1 | 100.0 |
| 16 | Esmedregal | 1 | 0 | 0.0 |
| 17 | Hamachi | 1 | 0 | 0.0 |
| 18 | Lenguado | 1 | 0 | 0.0 |
| 19 | Lisa | 1 | 0 | 0.0 |
| 20 | Mantarraya | 1 | 0 | 0.0 |
| 21 | Merluza | 1 | 0 | 0.0 |
| 22 | Pescado blanco | 1 | 1 | 100.0 |
| 23 | Xcochin | 1 | 0 | 0.0 |
|  | **Total** | **106** | **28** | **26.4** |
